# Prevalence of acute stress disorder among road traffic accident survivors: a meta-analysis

**DOI:** 10.1186/s12888-018-1769-9

**Published:** 2018-06-13

**Authors:** Wenjie Dai, Aizhong Liu, Atipatsa C. Kaminga, Jing Deng, Zhiwei Lai, Jianzhou Yang, Shi Wu Wen

**Affiliations:** 1 Department of Epidemiology and Health Statistics, Xiangya School of Public Health, Central South University, Changsha, Hunan China; 2 OMNI Research Group, Department of Obstetrics and Gynecology, Faculty of Medicine, University of Ottawa, Ottawa, ON Canada; 3 Ottawa Hospital Research Institute, Clinical Epidemiology Program, Ottawa, ON Canada; 4 School of Epidemiology, Public Health, and Preventive Medicine, Faculty of Medicine, University of Ottawa, Ottawa, ON Canada; 5 Department of Mathematics, Mzuzu University, Mzuzu, Malawi; 6 Immunization Programme Department, Hunan Provincial Center for Disease Control and Prevention, Changsha, Hunan China; 7 Department of Preventive Medicine, Changzhi Medical College, Changzhi, Shanxi China

**Keywords:** Acute stress disorder, Road traffic accident, Prevalence; meta-analysis

## Abstract

**Background:**

Road traffic accident (RTA), an unexpected traumatic event, may not only lead to death and serious physical injuries, but also could put survivors at an increased risk for a wide range of psychiatric disorders, particularly acute stress disorder (ASD). Early assessment of trauma-related psychological responses is important because acute trauma responses in the early post-traumatic period are among the robust predictors of long-term mental health problems. However, estimates of the prevalence of ASD among RTA survivors varied considerably across studies. Therefore, this meta-analysis aimed to identify the pooled prevalence of ASD among RTA survivors.

**Methods:**

A systematic literature search in the databases of PubMed, PsycINFO, PsycARTICLES, Embase and Web of Science was performed from their inception dates to December 2017. Subject headings were used to identify relevant articles, and the search strategy was adjusted across databases. Heterogeneity across studies was evaluated by Cochran’s *χ*^2^ test and quantified by the *I*^2^ statistic. Subgroup analyses were performed to identify the pooled prevalence in relation to the country of study, instrument used to identify ASD, age, gender and traumatic brain injury. When significant heterogeneity was observed, the influence of some potential moderators was explored using meta-regression analyses.

**Results:**

Thirteen eligible studies conducted in 8 countries were included. A total of 2989 RTA survivors were assessed, of which 287 were identified with ASD. The overall heterogeneity was high across studies (*I*^2^=96.8%, *P* < 0.001), and the pooled prevalence of ASD among RTA survivors was 15.81% (95% confidence interval: 8.27–25.14%). Subgroup analyses indicated that the prevalence of ASD among RTA survivors differed significantly with regard to the country of study, instrument used to identify ASD, age and gender (*P* < 0.05). Meta-regression analyses showed that mean age of participants and quality assessment score were significant moderators for heterogeneity (*P* < 0.05).

**Conclusions:**

Nearly one-sixth of RTA survivors suffer from ASD, indicating the need for regular assessment of early trauma responses among RTA survivors, as well as the importance of implementing early psychological interventions.

**Electronic supplementary material:**

The online version of this article (10.1186/s12888-018-1769-9) contains supplementary material, which is available to authorized users.

## Background

With the rapid increase in the number of motor vehicles in the past few decades, road traffic accidents (RTA) have become a public health problem [1–3]. According to the latest global status report on road safety by World Health Organization (WHO), more than 1.2 million people die from RTA each year, with millions more sustaining serious injuries and living with long-term adverse health consequences [http://www.who.int/violence_injury_prevention/road_safety_status/2015/en/]. Notably, children and adolescents are more vulnerable to RTA than adults, and the injuries caused by RTA is the leading cause of death among those aged 15 to 19 years and the second leading cause among those aged 10 to 14 years [http://www.who.int/violence_injury_prevention/child/injury/world_report/en/]. Accumulated evidence has shown that RTA, an unexpected traumatic event, may not only lead to death and serious physical injuries, but also could put survivors at an increased risk for a wide range of psychiatric disorders, particularly acute stress disorder (ASD) and post-traumatic stress disorder (PTSD) [4–6].

ASD, an acute trauma response that occurs within four weeks following a traumatic event, was first introduced into the fourth edition of the Diagnostic and Statistical Manual of Mental Disorders (DSM-IV) in 1994 [7]. According to the DSM-IV criteria, ASD is characterized by the symptom clusters of dissociation, intrusion, avoidance, and arousal [7]. The main difference between ASD and PTSD based on the DSM-IV criteria is the former’s emphasis on dissociative reactions to the trauma and the duration of the symptoms [8, 9]. Specifically, the ASD diagnosis requires that individuals must experience at least three dissociative symptoms, and PTSD can only be diagnosed from at least four weeks after the trauma, whereas ASD can be diagnosed from two days to four weeks after the trauma. A major reason for the introduction of ASD is to predict trauma-related individuals who would subsequently develop chronic PTSD [10]. In 2013, substantial changes have been made to the diagnosis of ASD with the release of DMS-V. In particular, ASD has been reclassified into Trauma- and Stressor-Related Disorders, and according to the DSM-V criteria, ASD is defined by five symptom clusters namely intrusion, negative mood, dissociation, avoidance, and arousal [11].

RTA is the most persistently common traumatic event in the modern world, and numerous studies have shown that ASD is quite prevalent among RTA survivors [12]. Estimates of the prevalence of ASD among RTA survivors varied considerably across studies, ranging from 1.6 to 41.1% [13–16], which may be mainly associated with the sample characteristics including gender, age, injury severity, and the instruments used to identify ASD [8, 13, 17]. Early assessment of trauma-related psychological responses is important because acute trauma responses in the early post-traumatic period are among the robust predictors of long-term mental health problems [18, 19]. For example, Bryant et al. found that 57% of males and 92% of females who met the criteria of ASD within one month following RTA were diagnosed with PTSD 6 months later [8], and Brewin et al. found that 83% of the victims of violent assaults who were diagnosed with ASD within 1 month following the crime still suffered from PTSD 6 months later [20]. In this regard, a reliable estimate of the pooled prevalence of ASD among RTA survivors is crucial, as it could help the health care providers determine an accurate amount of those who may develop ASD in the early period after RTA, and further estimate the amount of those who would subsequently suffer from long-term mental health problems. However, research interests have mainly focused on PTSD [21–23], and no meta-analysis has synthesized the evidence on the prevalence of ASD among RTA survivors to date. Therefore, this meta-analysis aimed to identify the pooled prevalence of ASD among RTA survivors.

## Methods

### Search strategy

This meta-analysis was performed according to the Preferred Reporting Items for Systematic Reviews and Meta-Analyses (PRISMA) guidelines (the Additional file 1). A systematic literature search in the databases of PubMed, PsycINFO, PsycARTICLES, Embase and Web of Science was performed from their inception dates to December 2017. Subject headings were used to identify relevant articles, and the search strategy was adjusted across databases. Specifically, for the database of PubMed and Web of science, search terms was: “Stress Disorders, Traumatic, Acute”[Mesh]) AND “Accidents, Traffic”[Mesh]; for the databases of Emabse and PsycARTICLES, search terms was: su(traffic accident) AND su(Acute Stress Disorder); and for the database of PsycINFO, a combination of the subject headings “Acute Stress Disorder” and “Motor Traffic Accidents” was applied. The reference lists of full articles were manually searched for more relevant publications.

### Eligibility criteria

Studies were included in this meta-analysis if they meet the following criteria: (1) the study design was observational; (2) the target population focused on or included RTA survivors; (3) the ASD diagnosis was made from two days to four weeks following RTA with specific reference to RTA; (4) the instrument used to identify ASD was based on the DSM-IV criteria with a binary outcome of “yes” or “no”;(5) information about the sample size and the prevalence of ASD among RTA survivors was provided; and (6) full article was written in English. Studies were excluded if they were abstracts, case reports, comments, reviews, or book chapters. Furthermore, if repeated data were observed across studies, the study published earlier was included.

### Data extraction

Two investigators independently assessed articles for eligibility and extracted relevant data from eligible articles. The primary outcome of this meta-analysis was the prevalence of ASD among RTA survivors, and for the purpose of this study, the following data were extracted: first author, year of publication, country of study, recruitment site, proportion of hospitalized participants, proportion of male participants, mean age of participants, instrument used to identify ASD, trauma-assessment interval, number of RTA survivors with ASD, and sample size. Consistent with previous meta-analyses exploring the post-traumatic stress responses among trauma-related populations [24, 25], the instruments used to identify ASD were categorized into self-report questionnaire and structured interview. Additionally, if available, data on traumatic brain injury (TBI) were extracted to perform subgroup analysis.

### Quality assessment

The Loney criteria, which has been widely used to evaluate observational studies estimating the prevalence of a health-related problem [26, 27], was used to assess the quality of the methodology of each eligible article for this meta-analysis. This instrument comprises eight items including (1) random sample or whole population, (2) unbiased sampling frame, (3) adequate sample size, (4) standard measures, (5) outcomes measured by unbiased assessors, (6) adequate response rate and refusers described, (7) confidence intervals (CI) and subgroups analysis, and (8) study subjects described. Each item is assigned a score of 1 point, and studies satisfying one item will be given 1 point. Thus, the total score of this instrument ranges from 0 to 8 points, with more scores indicating higher degree of quality.

### Statistical analysis

Statistical analyses were performed using SPSS 20.0 (IBM corp) and the “meta” and “metafor” package of R version 3.4.1. Heterogeneity across studies was evaluated by Cochran’s *χ*^2^ test and quantified by the *I*^2^ statistic, with *I*^2^ ≥ 25%, ≥ 50%, and ≥ 75% indicating low, moderate, and high heterogeneity, respectively [28]. The pooled prevalence of ASD among RTA survivors was combined using Freeman-Tukey double arcsine method by a random effects model if significant heterogeneity was observed across studies (*P* ≤ 0.10 and/or *I*^2^> 50%). Otherwise, a fixed effects model was used [29].

Subgroup analyses were carried out to identify the pooled prevalence according to some categorical study-level characteristics of the eligible studies. These included the country of study, instrument used to identify ASD (self-report questionnaire vs. structured interview), age (child or adolescent vs. adult), gender (male vs. female), and traumatic brain injury (yes vs. no). Differences across categories within each subgroup were compared using chi-square tests.

When significant heterogeneity was observed, mixed-model meta-regression analyses were conducted to explore the influence of some potential moderators on the heterogeneity using the restricted maximum-likelihood estimator method. The moderators tested were proportion of hospitalized participants, proportion of male participants, mean age of participants, instrument used to identify ASD, and quality assessment score of the eligible studies.

Sensitivity analysis was performed not only by serially removing each study, but also excluding low-quality studies to show their corresponding effects on the stability and strength of the pooled results [23, 30]. Publication bias was assessed by the Begg’s rank test, and a Begg’s funnel plot for asymmetry was presented. All statistical analyses were two-tailed with a significance level of 0.05.

## Results

### Search results

**Fig. 1:**
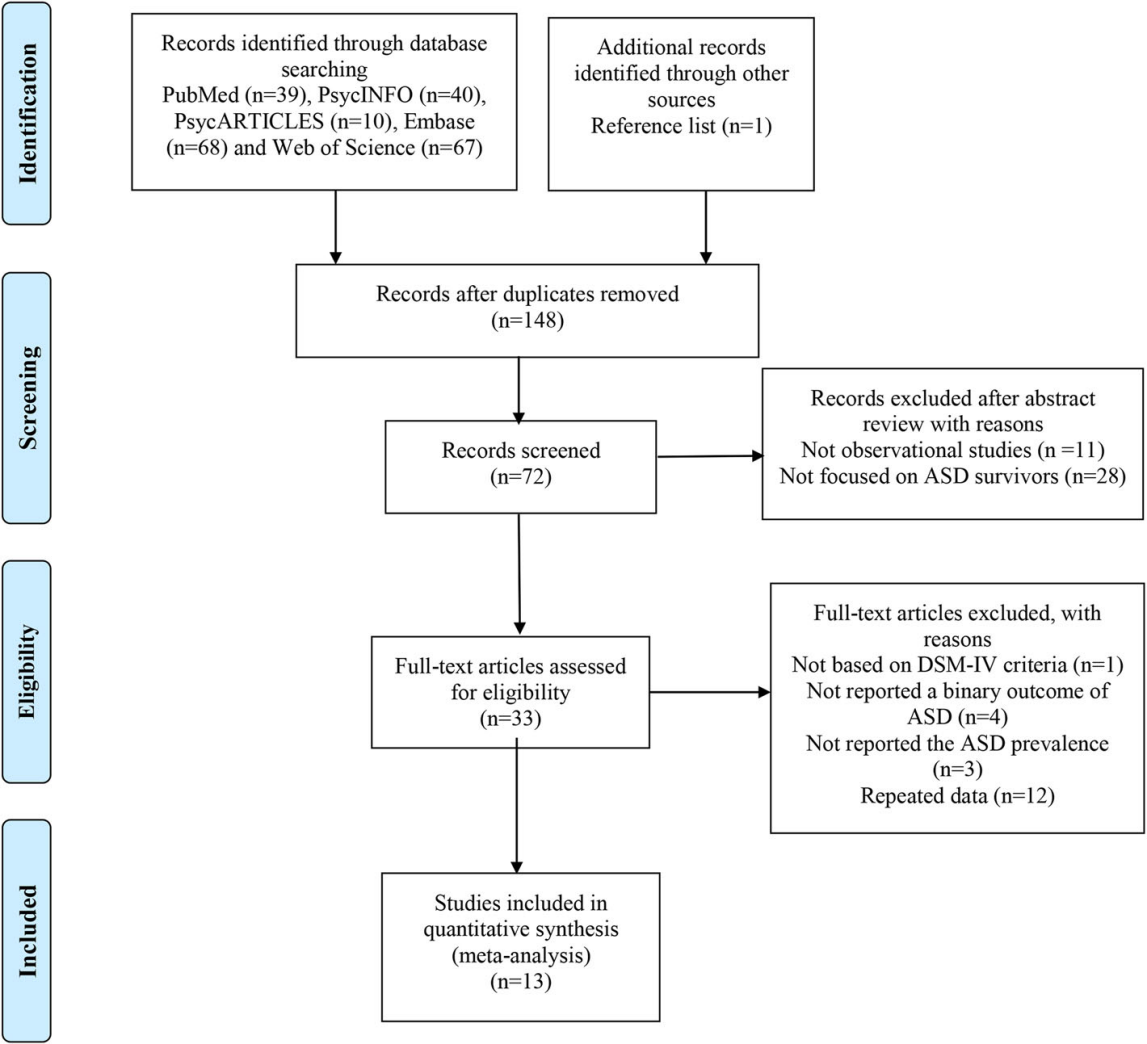
PRISMA flow chart of study identification process

A total of 225 articles were initially yielded. After excluding duplicates, 148 articles were screened. After reviewing abstract, 33 full articles were further shortlisted for eligibility assessment. Among the 33 full articles, 1 was excluded for not using the instrument based on DSM-IV criteria to identify ASD, 4 were excluded for not reporting a binary outcome of ASD, 3 were excluded for not reporting the prevalence of ASD, and 12 were excluded for repeating data. Finally, a total of 13 eligible articles were included in this meta-analysis (Fig. 1).

### Study characteristics

The characteristics of the eligible studies are shown in Table 1. The 13 studies were conducted in 8 countries: Australia, United Kingdom (UK), United States of America (USA), Denmark, Japan, Turkey, South Africa, and Switzerland. A total of 2989 RTA survivors were assessed, of which 287 were identified with ASD. Among the 13 eligible studies, 12 were hospital-based, and 1 was population-based; 5 focused on adults, and 6 focused on children or adolescents; 6 used exclusively self-report questionnaire to identify ASD, 6 used exclusively structured interview, and 1 used both self-report questionnaire and structured interview. Furthermore, ASD was assessed from 2 days to one month after RTA.

**Table 1:** Characteristics of the eligible studies

First author	Year of publication	Country of study	Recruitment site	Proportion of hospitalized participants, %	Proportion of male participants, %	Mean age (SD), years	Instrument used to identify ASD		Trauma- assessment interval	Number of survivors with ASD	Sample size
							Self-report questionnaire	Structured interview			
Bryant [46]	1998	Australia	Hospital-based	100.0	69.6	29.45 (12.62)	–	ASDI	2–28 days	11	79
Bryant [8]	2003	Australia	Hospital-based	100.0	63.7	Male: 29.95 (11.49) Female: 33.36 (13.28)	–	ASDI	Within 1 month	23	171
Fuglsang [52]	2004	Denmark	Hospital-based	6.7	45.6	33.99 (11.3)	ASDS	–	7–31 days	25	90
Jones [47]	2005	UK	Hospital-based	100.0	39.7	36.75 (12.77)	–	ASDI	Within 14 days	27	131
Hamanaka [53]	2006	Japan	Hospital-based	100.0	77.0	32.8 (14.5)	–	ASDI	Within 1 month	9	100
Yasan [14]	2009	Turkey	Hospital-based	100.0	70.5	33.93 (19.78)	–	SCID	4 days	39	95
van den Heuvel [54]	2016	South Africa	Hospital-based	NR	56.9	32.3 (10.6)	ASDS	–	10 days	47	115
Winston [55]	2002	USA	Hospital-based	100.0	72.0	10.4 (3.3)	CASQ	–	3–30 days	27	95
Bryant [56]	2004	UK	Hospital-based	21.0	55.0	12.27 (2.86)	Questions based on DSM-IV	–	2 weeks	13	86
Winston [13]	2005	USA	Population-based	NR	45.8	6.9	Questions based on DSM-IV	–	Within 30 days	24	1483
Salmon [15]	2007	Australia	Hospital-based	100.0	65.8	9.92 (2.55)	CASQ	–	6–28 days	6	76
Meiser-Stedman [16]	2007	UK	Hospital-based	100.0	68.1	11.88 (2.6)	Questionnaire based on DSM-IV	Interview based on DSM-IV	2–4 weeks	33	367
Haag [17]	2015	Switzerland	Hospital-based	61.4	58.4	11.55 (2.70)	–	IBS-A-KJ	10 days	3	101

*SD* standard deviation, *ASDI* Acute Stress Disorder Interview, *ASDS* Acute Stress Disorder Scale, *SCID* Structured Clinical Interview for DSM Disorders, *CASQ* Child Acute Stress Questionnaire, *IBS-A-KJ* Interview zur Akuten Belastungsstorung bei Kindern und Jugendlichen, *NR* not reported

The results of the methodological quality assessment of the eligible studies are shown in Table 2. Among the 13 eligible studies, 1 used random sampling method and was considered to have unbiased sampling frame. Additionally, 2 had a sample size of > 300 and all used established instruments to identify ASD. According to the Loney criteria, 1 scored 7 points, 9 scored 5 points and 3 scored 4 points.

**Table 2:** Methodological quality assessment of the eligible studies

Study	Random sample or whole population	Unbiased sampling frame	Adequate sample size (> 300 subjects)	Standard measures	Outcomes measured by unbiased assessors	Adequate response rate (> 70%) and refusers described	Confidence intervals and subgroups analysis	Study subjects described	Total score
Bryant et al. [46]	0	0	0	1	1	1	1	1	5
Bryant et al. [8]	0	0	0	1	1	1	1	1	5
Fuglsang et al. [52]	0	0	0	1	0	1	1	1	4
Jones et al. [47]	0	0	0	1	1	1	1	1	5
Hamanaka et al. [53]	0	0	0	1	1	1	1	1	5
Yasan et al. [14]	0	0	0	1	1	1	1	1	5
van den Heuvel et al. [54]	0	0	0	1	0	1	1	1	4
Winston et al. [55]	0	0	0	1	1	1	1	1	5
Bryant et al. [56]	0	0	0	1	0	1	1	1	4
Winston et al. [13]	1	1	1	1	0	1	1	1	7
Salmon et al. [15]	0	0	0	1	1	1	1	1	5
Meiser-Stedman et al. [16]	0	0	1	1	1	0	1	1	5
Haag et al. [17]	0	0	0	1	1	1	1	1	5

### Pooled prevalence of ASD among RTA survivors

**Fig. 2:**
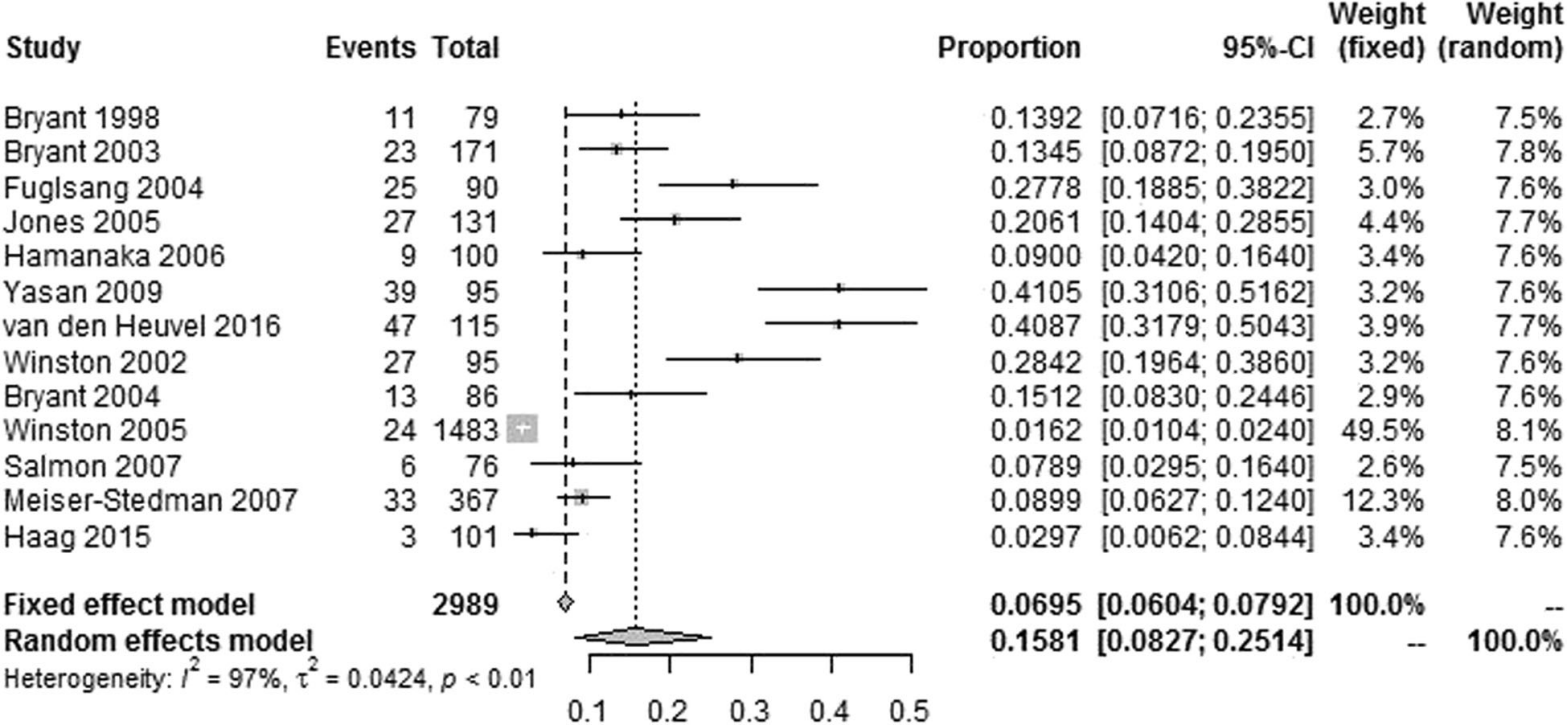
Forest plot of the eligible studies

The prevalence of ASD reported among the eligible studies varied from 3.0 to 41.1%, with the highest found among adult RTA survivors in Turkey and lowest found among child or adolescent RTA survivors in Switzerland [14, 17]. The overall heterogeneity was high across studies (*I*^2^=96.8%, *P* < 0.001), and the pooled prevalence of ASD among RTA survivors was 15.81% (95% CI: 8.27–25.14%) by a random effects model (Fig. 2).

### Subgroup analyses

The results of subgroup analyses are presented in Table 3. The pooled prevalence of ASD among RTA survivors in USA, UK, and Australia was 11.16% (95% CI: 0.00–48.57%), 14.24% (95% CI: 7.34–22.88%) and 12.11% (95% CI: 8.73–15.95%), respectively. The pooled prevalence of ASD among RTA survivors identified by self-report questionnaire and structured interview was 17.82% (95% CI: 4.05–38.11%) and 15.26% (95% CI: 7.03–25.81%), respectively. The pooled prevalence of ASD among child or adolescent RTA survivors and adult RTA survivors was 9.03% (95% CI: 2.90–17.89%) and 21.51% (95% CI: 11.82–33.08%), respectively. Additionally, the pooled prevalence of ASD among male and female RTA survivors was 7.43% (95% CI: 4.90–10.42%) and 17.89% (95% CI: 12.50–23.96%), respectively. Furthermore, the pooled prevalence of ASD among RTA survivors with and without TBI was 17.09% (95% CI: 11.30–23.75%) and 13.27% (95% CI: 3.69–27.13%), respectively. Also, the results of subgroup analyses indicated that the prevalence of ASD among RTA survivors differed significantly with regard to the country of study, instrument used to identify ASD, age and gender (*P* < 0.05).

**Table 3:** Subgroup analyses of the pooled prevalence of ASD among RTA survivors

Subgroup		Number of studies	Number of survivors with ASD	Total sample	Pooled prevalence (95% CI) (%)	Test of difference within each subgroup	
						Chi-square value	*P* value
Country of study						79.111	< 0.001^*^
	USA	2	51	1578	11.16 (0.00–48.57)		
	UK	3	73	584	14.24 (7.34–22.88)		
	Australia	3	40	326	12.11 (8.73–15.95)		
Instrument used to identify ASD						49.038	< 0.001^*^
	Self-report questionnaire	6	112	677	17.82 (4.05–38.11)		
	Structured interview	6	142	1945	15.26 (7.03–25.81)		
Age						170.088	< 0.001^*^
	Child or adolescent	6	106	2208	9.03 (2.90–17.89)		
	Adult	5	111	495	21.51 (11.82–33.08)		
Gender						13.667	< 0.001^*^
	Male	2	27	361	7.43 (4.90–10.42)		
	Female	2	32	177	17.89 (12.50–23.96)		
TBI						0.779	0.377
	Yes	2	25	145	17.09 (11.30–23.75)		
	No	2	19	141	13.27 (3.69–27.13)		

^*^P < 0.05

### Meta-regression analyses

The results of meta-regression analyses are presented in Table 4. The proportion of hospitalized participants, the proportion of male participants, and instrument used to identify ASD were not significant moderators for heterogeneity (*P* > 0.05). Mean age of participants and quality assessment score were significant moderators for heterogeneity (*P* < 0.05). Specifically, mean age of participants accounted for 32.40% of the heterogeneity and quality assessment score accounted for 36.80% of the heterogeneity across studies.

**Table 4:** Meta-regression analyses of the effects of potential moderators

	Number of studies	Coefficient	Standard error	*Z* value	*P* value	tau^2^
Proportion of hospitalized participants, %	11	−0.030	0.139	−0.217	0.828	0.021
Proportion of male participants, %	13	0.101	0.464	0.217	0.828	0.033
Mean age of participants, years	13	0.009	0.004	2.467	0.014^*^	0.020
Instrument used to identify ASD	13	−0.046	0.103	−0.455	0.649	0.032
Quality assessment score	13	−0.142	0.054	−2.661	0.008^*^	0.019

^*^P < 0.05

### Sensitivity analysis and publication bias

After one-by-one removals of 13 studies, the pooled prevalence of ASD among RTA survivors varied from 14.09% (95% CI: 7.27–22.61%) to 17.66% (95% CI: 11.28–25.08%), and the *I*^2^ statistic varied from 91.7 to 97.1%. Specifically, after removing one study which used both the self-report questionnaire and structured interview to identify ASD, the pooled prevalence of ASD was 16.50% (7.77–27.58%) and the *I*^2^ statistic was 96.8%. Additionally, after excluding one population-based study, the pooled prevalence of ASD was 14.90% (7.44–24.28%), and the *I*^2^ statistic was 97.1%. Moreover, after removing three studies with quality assessment score of 4, the pooled prevalence decreased to 12.85% (95% CI: 5.94–21.79%), and the *I*^2^ statistic was 96.4%.

**Fig. 3:**
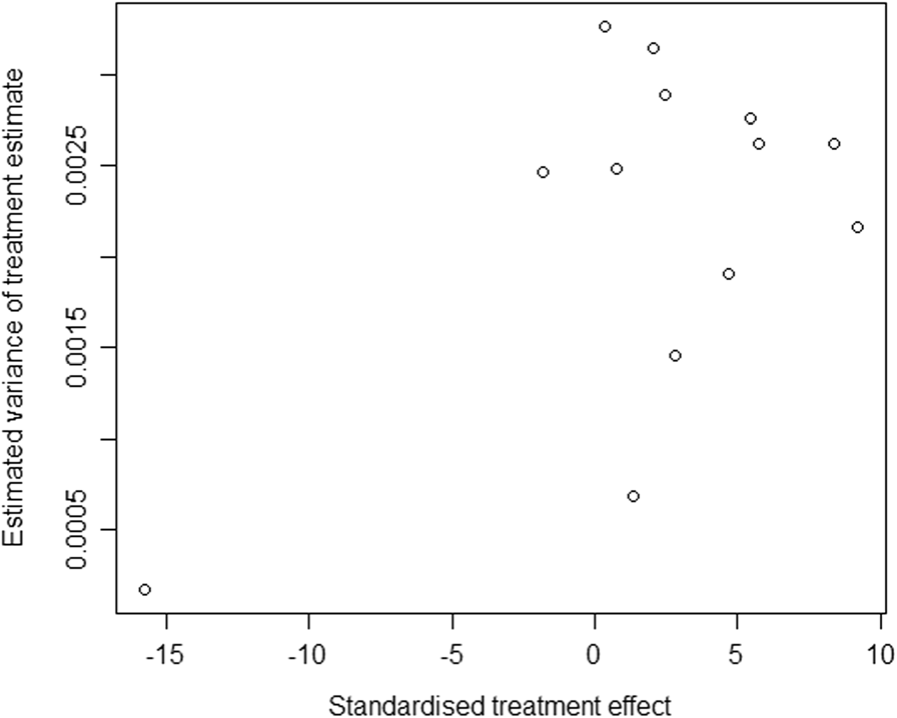
Begg’s funnel plot of the eligible studies

Publication bias was not observed in this meta-analysis, with *P* value for the Begg’s rank test being 0.760 (z = 0.306). A Begg’s funnel plot is presented in Fig. 3.

## Discussion

This meta-analysis synthesized the evidence on the prevalence of ASD among RTA survivors. Thirteen eligible studies conducted in 8 countries were included. A total of 2989 RTA survivors were assessed, of which 287 were identified with ASD. Results showed that the pooled prevalence of ASD among RTA survivors was 15.81% (95% CI: 8.27–25.14%). To the best of our knowledge, this study provides the first quantitative estimate of the pooled prevalence of ASD among RTA survivors.

The pooled prevalence of ASD among RTA survivors (15.81, 95% CI: 8.27–25.14%) found in this study was lower than the prevalence of ASD found among earthquake survivors (28.4%), victims of major burn injuries (23.6%), and victims of physical assault (24%) [31–33]. However, it was much higher than the prevalence of ASD among myocardial infarction patients (3.6%) and victims of typhoon (7.2%) [34, 35]. Given the rapid increase in the occurrence of RTA worldwide and the high pooled prevalence of ASD among RTA survivors found in this study, health care providers should pay more attention to the assessment of early trauma responses among RTA survivors and implement early psychological interventions accordingly.

A significant difference in the pooled prevalence of ASD among RTA survivors across countries was observed by subgroup analyses, which could be mainly associated with cultural difference across countries. For example, it has been well-established that the profile of ASD symptoms varies greatly across cultures, especially with regard to the symptom cluster of dissociation [36]. Furthermore, the disparity of genetic background across ethnicities may have played a role in the different pooled prevalence observed across countries [37].

In terms of the instruments used to identify ASD, structured interview is the “gold standard” to diagnose ASD, while some self-report questionnaires with high specificity and sensitivity, such as the ASDS, have been widely used when structured interview was not feasible [38]. Numerous studies have shown that compared with structured interview, self-report questionnaires tend to overestimate the prevalence of some psychiatric disorders [39, 40]. For example, Swartzman et al. synthesized the results of 11 studies and found that the pooled prevalence of PTSD among cancer patients assessed using structured interview was 4.0% (95% CI:2.6–6.2%), while that assessed using self-report questionnaire was 12.8% (95% CI: 10.8–15.0%) [40]. Consistently, this study found that the pooled prevalence of ASD among RTA survivors identified by self-report questionnaires (17.82, 95% CI: 4.05–38.11%) was significantly higher than that identified by structured interviews (15.26, 95% CI: 7.03–25.81%), which indicated that caution should be applied when using self-report questionnaires to identify ASD among RTA survivors.

Subgroup analyses also found that the pooled prevalence of ASD was significantly higher among female RTA survivors. Gender difference in the pooled prevalence of ASD could be attributed to the gender difference in the coping strategy and interpretation of the trauma. Specifically, compared with men, women interpret trauma more stressfully and are more likely to take negative coping strategies when exposed to a trauma [41]. Additionally, women could report their post-traumatic response more easily than men [42].

The prevalence of ASD among child and adolescent RTA survivors was estimated to be low in many studies [13, 17], and our study found that the pooled prevalence of ASD was significantly higher among adult RTA survivors than child or adolescent RTA survivors. Also, meta-regression analyses indicated that the mean age of the participants was a significant source of heterogeneity. The profile of ASD symptoms is different between adults and children or adolescents. In particular, the occurrence of the dissociative symptom on the DSM-IV criteria was quite low among children and adolescents. For example, Meiser-Stedman et al. found that the symptom cluster that prevented a full ASD diagnosis among child and adolescent was overwhelmingly (73.5%) the dissociative criterion [16].

Numerous studies have consistently shown that moderate to severe TBI could lead to negative alterations in cognition, and individuals who sustained TBI exhibit more severe post-traumatic stress symptoms [43–45]. However, though this study found that the pooled prevalence of ASD was higher among those with TBI than those without TBI, the difference was not significant, which could be explained by the fact that the severity of TBI among RTA survivors who were able to participate in studies conducted at an early period after trauma was mostly mild [46, 47]. Additionally, it’s worth noting here that only 2 eligible studies reported relevant data when estimating the pooled prevalence of ASD in relation to TBI. Therefore, more studies are needed to clarify the association between TBI and ASD among RTA survivors.

The result of sensitivity analyses indicated that the quality of study may affect the stability of the pooled results. After excluding 3 studies with relatively low quality, the pooled prevalence decreased greatly from 15.81% (95% CI: 8.27–25.14%) to 12.85% (95% CI: 5.94–21.79%). Also, meta-regression analyses showed that quality assessment score was a significant moderator for heterogeneity. Generally, studies with low quality are more likely to apply biased sampling frame with small sample size, and thus tend to overestimate the effect size [48, 49]. Therefore, more studies with unbiased sampling frame and large sample size are warranted to obtain a more reliable estimate.

This study has several limitations. First, this meta-analysis included exclusively studies which identified ASD using the instruments according to the DSM-IV criteria, which may preclude generalizing the findings of this study to studies identifying ASD according to the DSM-V criteria. Second, though evidence has shown that history of prior trauma, history of prior psychiatric disorders, and perceived social support level may be related to post-traumatic stress symptoms [14, 50, 51], subgroup analyses according to these factors were unable to be conducted since few studies reported relevant information. Additionally, subgroup analyses were carried out without adjustment for potential confounders.

## Conclusions

The pooled prevalence of ASD among RTA survivors is 15.81% (95% CI: 8.27–25.14%) and varies significantly with regard to the country of study, instrument used to identify ASD, gender and age. Mean age of participants and quality assessment score were significant moderators for heterogeneity. The high pooled prevalence of ASD among RTA survivors underscores the importance of regular assessment of early trauma responses among RTA survivors, and the implementation of effective psychological interventions is recommended.

## Additional file

Additional file 1: Details of PRISMA 2009 Checklist. (DOC 64 kb)

## Abbreviations

ASD: Acute stress disorder
ASDI: Acute stress disorder interview
ASDS: Acute stress disorder scale
CASQ: Child acute stress questionnaire
CI: Confidence interval
DSM: Diagnostic and statistical manual of mental disorders
IBS-A-KJ: Interview zur Akuten Belastungsstorung bei Kindern und Jugendlichen
NR: Not reported
PRISMA: Preferred reporting items for systematic reviews and meta-analyses
PTSD: Post-traumatic stress disorder
RTA: Road traffic accidents
SCID: Structured clinical interview for DSM disorders
SD: Standard deviation
TBI: Traumatic brain injury
WHO: World health organization
